# Shaping outcome of ProTaper NEXT for root canal preparation in mandibular incisors: a micro-CT study

**DOI:** 10.1186/s12903-022-02335-7

**Published:** 2022-07-22

**Authors:** Hao Wang, Xueqin Yang, Ling Zou, Dingming Huang, Xuedong Zhou, Jialei Xu, Yuan Gao

**Affiliations:** 1 State Key Laboratory of Oral Diseases, National Clinical Research Center for Oral Diseases, Department of Cariology and Endodontics, West China Hospital of Stomatology, Sichuan University, 14#, 3Rd Section of RenMin South Road, Chengdu, 610041 People’s Republic of China; 2 Department of Stomatology, Shenzhen Longhua District Maternity and Child Healthcare Hospital, Shenzhen, 518000 People’s Republic of China; 3 Key Laboratory of Oral Biomedical Research of Zhejiang Province, The Affiliated Hospital of Stomatology, School of Stomatology, Zhejiang University School of Medicine, Hangzhou, Zhejiang 310006 People’s Republic of China

**Keywords:** Dentin thickness, Mandibular incisors, Micro-CT, Root canal preparation, Untouched canal wall

## Abstract

**Background:**

Relatively high incidence of single canals with oval or round shape were observed in human mandibular incisors. In order to investigate the influence of the root canal morphology on root canal preparation, the shaping outcome of ProTaper NEXT in oval and round canals of mandibular incisors were evaluated by using micro-computed tomography (micro-CT) analysis.

**Methods:**

This experiment was approved by the School Medical Ethics Committee. The sample size calculation was conducted using G*Power software. Intact mandibular incisors with a single canal were selected. Oval canals (2 < aspect ratio (AR) ≤ 4) and round canals (AR ≤ 2) were pair-matched according to canal length, and assigned to two experimental groups (13 per group). ProTaper NEXT was used for root canal preparation for both groups. Untouched canal wall (UCW), root canal morphological parameters and three-dimensional (3D) dentin thickness were evaluated in the entire root canal and each canal third after micro-CT scanning. Statistical analysis: Data were collected and analyzed with Mann–Whitney test and Friedman test using SPSS statistics software 25 (*P* < 0.05). Additionally, correlations of UCW area with canal morphological parameters were also investigated.

**Results:**

After root canal preparation, no significant difference was observed in all analyzed parameters in the apical third between oval and round canal groups (*P* > 0.05). In the coronal two thirds of the canal, the post-operative structure model index (SMI), form factor and roundness were significantly increased, while the AR was significantly decreased in both groups (*P* < 0.05). In addition, in the coronal two thirds, significantly more UCW and higher UCWΔ% was observed in oval canal group (*P* < 0.05). Furthermore, UCW correlated very strongly to canal major diameter (0.924) and initial volume (0.938), and strongly to canal form factor (− 0.724), minor diameter (0.799) and canal area (0.882). Proximal dentin wall was associated with significantly thinner pre-operative dentin thickness and higher amount of dentin removal after root canal preparation in both oval and round canal groups.

**Conclusions:**

(1) Both types of canals were more conical after root canal preparation, but oval root canals tend to leave more UCW area than round canals in the coronal two thirds of mandibular incisors, which contributes to the challenge for endodontic infection control. (2) Root canal preparation usually results in excessive dentin removal in the proximal dentin wall comparing with buccal and lingual walls in both types of canals of mandibular incisors.

**Supplementary Information:**

The online version contains supplementary material available at 10.1186/s12903-022-02335-7.

## Background

The main goal of root canal preparation is to remove the inner layer of infected dentin and eradicate bacterial populations or at least reduce them to levels that allow for periapical tissue to heal [1, 2]. In previous studies, different methods including measuring microscope and micro-CT were used to evaluate the incidence of oval canals in mandibular incisors in different human populations. Relatively high incidence (32.4–72%) were observed in 3–7 mm from the apex [3–5]. It has been widely recognized that effective root canal preparation is a challenging task when encountering oval or flattened-shaped root canals in mandibular incisors because they present buccal and lingual extensions that are difficult to access [6].

Multiple studies have focused on the outcomes to determine the shaping ability of different files in oval canals [7–12]. Zuolo et al. investigated the potential effects of root canal morphology on the shaping outcome of NiTi preparation by using micro-CT. In this study, the shaping ability of four root canal preparation systems were compared in oval-shaped canals of mandibular incisors. It was revealed that none of the tested instruments was able to completely shape the canals, and left certain amount of untouched canal wall (UCW) after canal shaping [7]. The UCW area ranged from 3.9 ± 1.8% to 58.8 ± 8.5% among previous studies [7–18]. In the study of Siqueira Junior et al., the microscope examination found that UCW was covered with bacteria and/or tissue debris in the majority of specimens [6]. The persistence of bacteria could influence the treatment outcome when a stable nutrient source is established [6]. Another concern related to ineffective canal shaping was the potential influence on the canal obturation quality [19].

Most previous studies of mandibular incisors focused on oval-shaped canals, either evaluated the performance of newly developed files or compared the outcome of different files [7–17, 20–24]. As far as we know, there has been no study focused on comparing the different influence of root canal morphology (both oval and round root canals) of mandibular incisors on the shaping outcome of root canal preparation systems by using micro-CT.

According to Perez’s study [21], preparing the mandibular incisor root canal to a larger size can significantly reduce the UCW. However, a significant decrease in dentin thickness was also observed after a progressively larger preparation. For tooth survival, it is essential to preserve dentin so as not to weaken the tooth [24]. To obtain 1 mm of dentin thickness in the mesio-distal direction is recommended in several previous studies [25–28].

Most studies evaluated the dentin thickness in mandibular incisors after root canal preparation only used 2-dimensional parameters, evaluating dentin thickness on cross-section images [21, 23, 29]. However, the dentin thickness on certain level of root cannot represent the overall condition because of the anatomic complexity of the root canal system. Micro-CT has been widely used in dentin thickness studies as it provides nondestructive and high-precision 3-dimensional (3D) anatomical and geometric information of the teeth [30–33]. These studies mainly evaluated dentin thickness in mandibular premolars and molars. The 3D measurement of dentin thickness after root canal preparation in mandibular incisors has not been reported yet.

Endodontic instruments have improved after undergone a series of modifications in design and material. The ProTaper Next system (Dentsply Maillefer, Ballaigues, Switzerland) owns an innovative asymmetric characteristic which permits only two cutting edges touch the canal wall under a continuous rotation [34]. This system is made from NiTi M-Wire and exhibits better mechanical properties than conventional NiTi instruments [14]. Micro-computed tomography imaging technology has been considered the gold standard to study the shaping ability of instruments because it provides a 3D high-resolution non-destructive analysis of the inner structure of teeth [14].

Considering all the mentioned points, the aim of this study was to evaluate the effects of root canal morphology on the shaping outcome of ProTaper NEXT in mandibular incisors by using micro-CT. The null hypothesis tested was that the root canal morphology (oval or round root canal) would not influence the outcome of root canal preparation.

## Materials and methods

### Selection of teeth

This study was approved by the Medical Ethics Committee of West China Stomatology Hospital, Sichuan University (WCHSIRB-D-2020-388). Forty-three mandibular incisors that had been extracted for reasons unrelated to this study were collected. The inclusion criteria were as follows: (1) teeth with one single canal, (2) no significant calcification or internal resorption defects, (3) no significant external root defects. Teeth with cracks, immature apices, root external defects, or coronal fillings were excluded from this study. Each tooth was scanned in a micro-CT scanner (μCT-50; Scanco Medical, Bassersdorf, Switzerland). The scanner parameters were set as 90 kV, 88μA, 8 W, resulting in an image with a 24 μm voxel size. The intracanal anatomy was confirmed according to the micro-CT scanning.

### Root canal preparation

All root canal preparation were conducted by a single experienced clinician. The clinician was not aware of grouping and was not allowed to see the micro-CT scans until all specimens were completely prepared. A conventional access cavity was prepared using highspeed diamond burs following guidelines described in the literature. The initial entry was on the lingual surface of the crown, and the cavity was extended until the complete removal of the pulp chamber roof [15]. No coronal flaring was performed. The canals were initially scouted with a size 10 K-file (Dentsply Sirona Endodontics) until its tip was visible at the apical foramen, and the working length (WL) was set 0.5 mm shorter. Apical patency was achieved with a size 15 K-file. Teeth were then prepared with ProTaper Next system. The sequence of ProTaper NEXT instruments were: X1, X2, X3. Each instrument was passively introduced into the root canal at a 300 rpm rotation rate and 2.5 N/cm torque driven by the endodontic motor according to manufacture guideline. Between successive steps, they were irrigated with 2 mL of 2.5% NaOCl. A final flush with 5 mL distilled water was inserted into the root canal and ultrasonically activated for 1 min [7].

### Micro-CT imaging

After root canal preparation, the samples were re-scanned with the aforementioned parameters. Both pre-operative and post-operative data were exported as DICOM files. The DICOM files were registered with Elastix rigid image registration module within 3D Slicer v4.1.1 software (Harvard SPL, Boston, MA, USA), based on image intensity similarities with the precision of better than 1 voxel. The region of interest (ROI) was selected to extend from the cemento-enamel conjunction to the most coronal slice showing the apical foramen using CTAn v1.18.8 software (Bruker, Billerica, Commonwealth of Massachusetts, USA).

Pre-operative canal AR was measured using CTAn software and ImageJ software (National Institutes of Health, Bethesda, MD). According to the pre-operative value of aspect ratio (AR) in coronal two thirds of the root canal, teeth were defined as oval canals (2 < AR ≤ 4) and round canals (AR ≤ 2) [35]. Each tooth with round-shaped canal was pair-matched with another tooth with oval-shaped canal according to ROI root canal length. Teeth without suitable match were excluded. Finally, 5 teeth were excluded for not fulfilling the inclusion criteria, 1 tooth with round canal and 11 teeth with oval canals were excluded for not having a suitable match.

The sample size calculation was based on previous studies on root canal preparation efficacy [36–41]. These studies typically assessed 6–30 canals per group and reported differences in the proportion of UCW ranging from 4 to 100%. The sample size calculation was conducted in G*Power 3.1.9.7 software using the following formula: “The ANOVA: Fixed effects, omnibus, one-way was selected from the F-test family” with 80% power and 5% significance. A sample size of 18 teeth (9 per group) was indicated as the minimum to reveal statistical significance among groups. A total of 26 teeth (13 per group) were acquired and were allocated into either round canal group or round canal group.

### Micro-CT measurement

#### Root Canal morphological analysis

The cross-section image slices of ROI of pre- and post-operative mandibular incisors were acquired before and after root canal preparation, and then divided into apical, middle and coronal thirds. From these semi-auto-segmented image stacks, the following root canal morphological parameters were measured using CTAn software and ImageJ software (National Institutes of Health, Bethesda, MD): structure model index (SMI), form factor, roundness, AR, major diameter, minor diameter, root canal area and root canal volume [4, 5]. The percentage change of each parameter (%Δ) was calculated by using pre-operative (PRE) and post-operative (POST) values, according to the formula:

$$\% \Delta = \left( {{\text{PRE}} - {\text{POST}}} \right)/{\text{PRE}}*{1}00\%$$

Matched images of the surface areas of the pre- and post-operative root canals were examined to evaluate the percentage of UCW surface with MeVislab v3.2 software (MeVis Medical Solutions AG, Bremen, Germany) and Geomagic studio software (Raindrop Geomagic, Research Triangle Park, NC) [24]. The pre- and post-operative canal surfaces were imported into Geomagic Studio software. The post-operative canal surface was thickened by one voxel (24 μm in this case) in both positive and negative directions. According to the detailed technical method note of the UCW measurement with micro-CT [42], the pre-operative canal surface which coincides in space with the 2 voxel-thick post-operative canal surface, will be considered as UCW, which are not changed by the preparation procedure. Accordingly, apply the intersect option between the pre-operative canal surface and the thickened post-operative surface model, then acquire the area of UCW [42].

All analyses were conducted in the entire canal and in each third of the root canal (apical, middle and coronal). The percentage of UCW was calculated by using post-operative UCW area value and the entire pre-operative root canal area, according to the formula:

$$\% {\text{UCW}} = {\text{UCW}}\;{\text{area}}/{\text{pre}} - {\text{operative}}\;{\text{canal}}\;{\text{area}}*{1}00\%$$

The Spearman correlation analysis was used to verify the correlations of UCW with root canal morphological parameters. Correlation coefficient of 0.7–0.9 indicates a strong correlation between pairs, whilst coefficient of 0.9–1.0 reveals a very strong correlation [43].

#### 3D dentin thickness analysis

Dentin thickness of the coronal 2/3 of the root were investigated by using Mevislab software and Geomagic Studio software. The pre-operative and post-operative root canal surfaces, as well as the external root surfaces were segmented and generated using Mevislab software, exported as STL files. Then they were imported into Geomagic studio software to extract the external root surfaces (buccal, lingual and proximal), as well as the root canal surfaces. These surfaces were imported into MeVisLab software afterwards for auto-calculation of the dentin thickness in different directions (buccal, lingual and proximal) by searching for the opposite surface at each point. The shortest distance between the external root surfaces and the root canal surfaces was selected as the minimum dentin thickness. Meanwhile, the mean dentin thickness was also generated automatically.

Pre- and post-operative mandibular incisors of the coronal 2/3 of the root were used to investigate the 3D dentin thickness distribution. The mean and minimum pre-operative dentin thickness (PRE-DT) and remaining dentin thickness (RDT) after root canal preparation in different directions of each tooth were obtained. Based on these values, the percentage of decrease in dentin thickness was calculated according to the formula:

$$\% \Delta {\text{dentin}}\;{\text{thickness}} = \left( {{\text{PRE}} - {\text{DT}} - {\text{RDT}}} \right)/{\text{PRE}} - {\text{DT}}*{1}00\%$$

An examiner blinded to the grouping protocols performed the analysis. The schematic illustration of the overall methods was presented in Additional file 1: Fig. S1.

Morphological parameters evaluated in this study were as following:

- *Structure model index (SMI)* Structure model index indicates the relative prevalence of rods and plates in a 3D structure such as trabecular bone. It involves a measurement of surface convexity in a 3-dimensional structure [44].
- *Form factor* Form factor is a 2D morphometric parameter, calculated by the equation: (4*π*A)/Pm^2^, where A and Pm are object area and perimeter respectively.
- *Roundness* Roundness is calculated by the equation: 4*A*/(π*d_max_)^2^), where *A* is the object area. The value of *R* ranges from 0 to 1, with 1 signifying a circle.
- *AR* Aspect ratio is defined as the ratio of maximum diameter to minimum diameter.

### Statistical analysis

The statistical analysis between groups was performed with the Mann–Whitney test. The Friedman test was used for within group analysis. The significance level was set at 0.05. SPSS statistics software (Version 25; IBM SPSS, Inc, Chicago, IL, USA) was used to carry out statistical analysis.

## Results

**Fig. 1:**
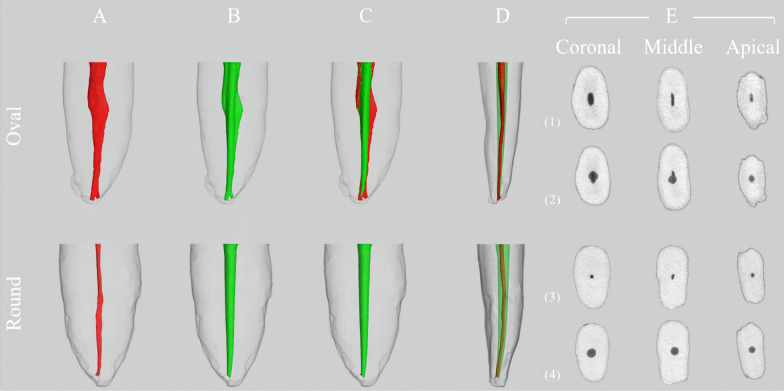
The representative 3-D models of round and oval canal in mandibular incisors. **A** Oval and round canal model before root canal preparation. **B** Oval and round canal after root canal preparation. **C** Matched and superimposed root canal indicated the untouched surface (red) and touched surface (green) from mesio-distal view. **D** Matched and superimposed root canal indicated the alteration of canal geometry after preparation, pre-operative canals (red) and the post-operative canals (green) from buccal-lingual view. **E** Cross-sectional views of the coronal, middle and apical thirds of before and after root canal preparation

The 3D models of round canals and oval canals in mandibular incisors before and after root canal preparation was constructed and visualized (Fig. 1).

### Root canal morphological parameters

In the apical third, no significant difference was observed in pre-operative root canal morphological parameters (SMI, form factor, roundness, AR, major diameter, minor diameter, canal area and canal volume) between oval and round canal groups (Additional file 2: Fig. S2-A3-H3, Additional file 4: Table S1). In the coronal and middle thirds, significant difference was observed in pre-operative root canal geometric parameter (SMI, form factor, roundness, AR, major diameter, canal area, canal volume) between oval and round canal groups (*P* < 0.05) (Additional file 2: Fig. S2-A1-H1, A2-H2, Additional file 4: Table S1).

**Fig. 2:**
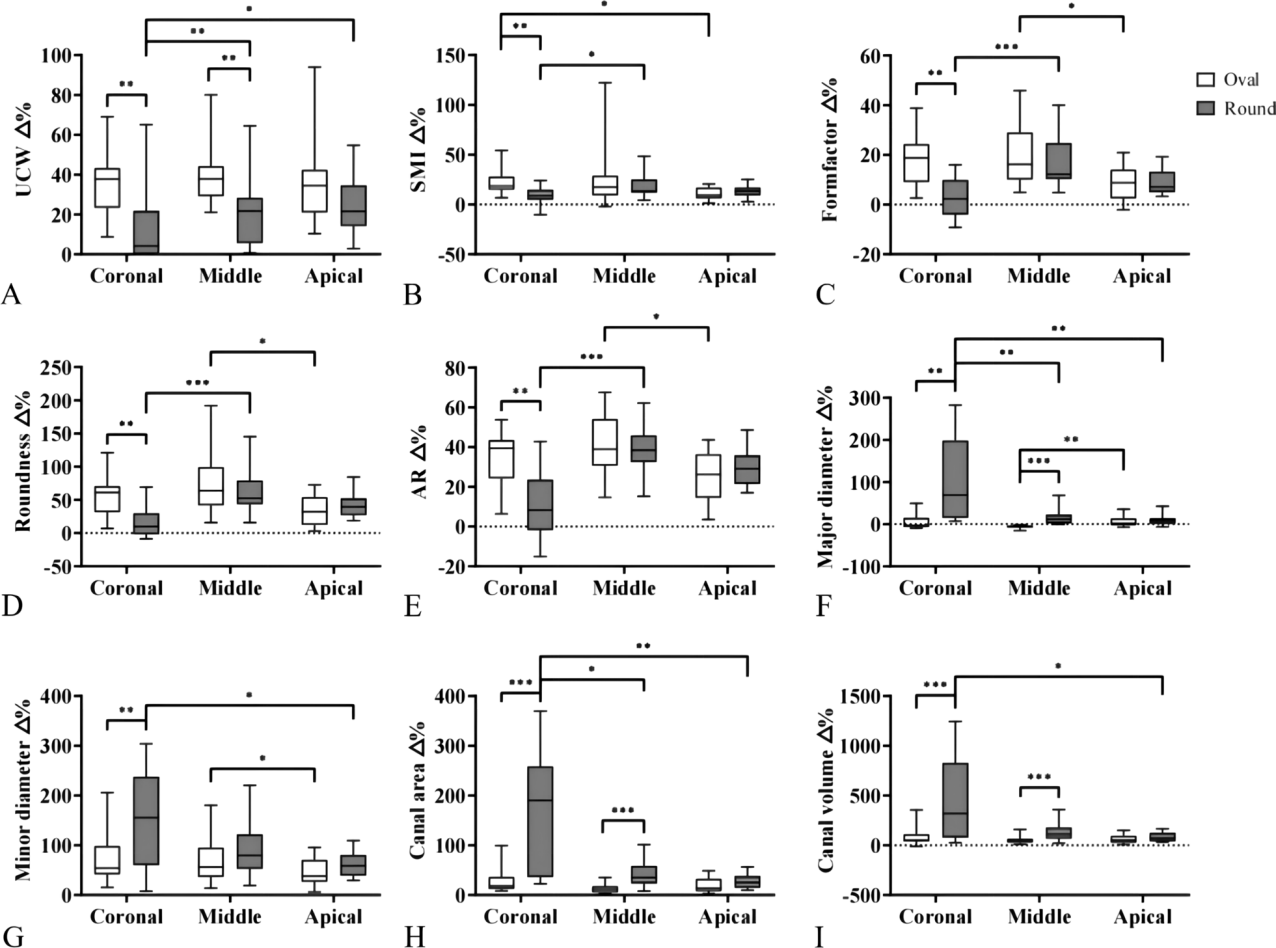
Percentage change of the root canal morphological parameters after root canal preparation. The percentage change of UCW (**A**), SMI (**B**), Formfactor (**C**), Roundness (**D**), AR (**E**), major diameter (**F**), minor diameter (**G**), canal area (**H**) and canal volume (**I**) were evaluated in both oval canal group and round canal group in different regions (coronal, middle and apical third). *indicates *P* < 0.05, **indicates *P* < 0.01, ***indicates *P* < 0.001. UCW: Untouched canal wall. SMI: Structure model index. AR: aspect ratio. Δ%: the percentage change of the canal morphological parameters

After root canal preparation, no significant difference was observed in all analyzed parameters in the apical third between oval and round canal groups (*P* > 0.05). In the coronal third and middle third, the values of SMI, form factor, roundness significantly increased in both oval root canal and round canal groups (*P* < 0.05), while the AR significantly decreased (Additional file 2: Fig. S2, Additional file 4: Table S1). Post-operative major diameter and canal area were found significantly different between oval and round canal groups in the middle third (*P* < 0.05), but not in the coronal third (Additional file 2: Fig. S2-E1, E2, G1, G2). Significant difference in the change percentage of all root canal morphological parameters between oval canal and round canal group was observed in coronal and middle thirds (Fig. 2).

More UCW and UCWΔ% were left in oval canal group comparing with round canal group after root canal preparation (*P* < 0.05). What’s more, more UCW and UCWΔ% were observed in coronal and middle thirds of oval canal group (*P* < 0.05) (Fig. 2A, Additional file 4: Table S1). UCW correlated very strongly to canal major diameter (0.924) and initial volume (0.938), and strongly to canal form factor (− 0.724), minor diameter (0.799) and canal area (0.882) (Table 1).

**Table 1:** The correlation coefficients (R) of each root canal morphological parameters and UCW

	UCW (R)
Canal area	0.882*
Canal volume	0.938**
SMI	− 0.406
Roundness	− 0.626
Formfactor	− 0.724*
Major diameter	0.924**
Minor diameter	0.799*

*Indicates a strong correlations (0.7–0.9),

**Indicates a very strong correlation (0.9–1.0) between the root canal morphological parameters and UCW

### 3D dentin thickness distribution

**Fig. 3:**
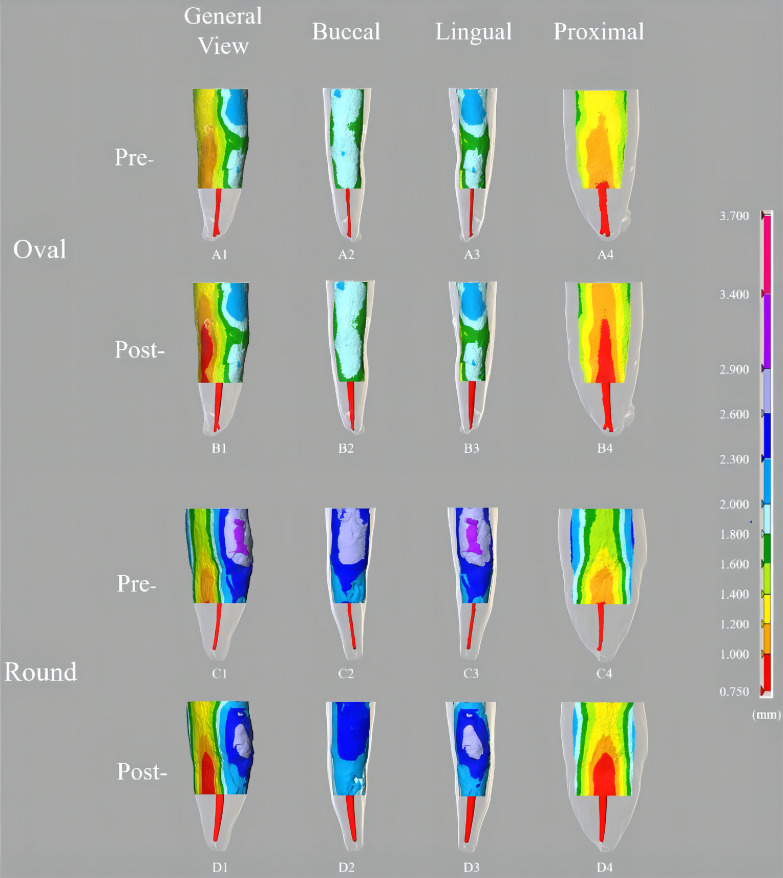
Color-coded 3D distribution of dentin thickness. Color-coded 3D distribution of dentin thickness between internal canal surface to external root surface in coronal 2/3 of the mandibular incisors on different surface before (pre-) and after root canal preparation (post-) were visualized. (**A1**, **B1**, **C1**, **D1**) represent the general view of the thickness distribution from 45° view. The thickness distribution of root 2/3 on the buccal root surface (**A2**, **B2**, **C2**, **D2**), the lingual root surface (**A3**, **B3**, **C3**, **D3**), and the proximal root surface (**A4**, **B4**, **C4**, **D4**). Pre-: Pre-operative model. Post-: Post-operative model

According to the study of Silva et al., 1.3 mm of remaining dentin thickness is a critical point relating to the resistance of teeth [29]. Before root canal preparation, the dentin thickness of most of the teeth evaluated in this study (92.3%) was over 1.3 mm in the coronal third. In the middle third, the average dentin thickness of proximal walls (1.35 ± 0.22 mm) was much thinner than the buccal (1.95 ± 0.32 mm) and lingual walls (2.01 ± 0.35 mm) (Fig. 3, Table 2).

**Table 2:** The 3D dentin thickness analysis of the coronal 2/3 of the root in mandibular incisors

		Coronal third		Middle third	
		Oval	Round	Oval	Round
Mean buccal DT	Pre-	2.01 (1.87, 2.17)^a^	2.19 (1.98, 2.37)^a^	1.88 (1.78, 2.09)^a^	2.05 (1.90, 2.26)^a^
	Post-	1.98 (1.87, 2.17)^a^	2.06 (1.69, 2.21)^a^	1.85 (1.77, 2.08)^a^	1.96 (1.86, 2.21)^a^
	Δ%	0.00 (0.00, 0.70)^a^	7.25 (1.96, 11.81)^b^	0.58 (0.00, 1.74)^a^	2.22 (1.84, 4.26)^b^
Minimum buccal DT	Pre-	1.72 (1.55, 1.80)^a^	1.82 (1.72, 1.91)^a^	1.56 (1.45, 1.77)^a^	1.68 (1.50, 1.76)^a^
	Post-	1.72 (1.55, 1.79)^a^	1.65 (1.30, 1.84)^a^	1.48 (1.44, 1.76)^a^	1.63 (1.43, 1.6)^a^
	Δ%	0.00 (0.00, 0.60)^a^	10.26 (0.60, 18.30)^b^	1.18 (0.00, 5.22)^a^	3.55 (0.91, 5.93)^a^
Mean lingual DT	Pre-	2.20 (2.03, 2.43)^a^	2.40 (2.23, 2.60)^ac^	1.89 (1.77, 2.24)^a^	2.09 (2.00, 2.38)^a^
	Post-	2.07 (1.97, 2.30)^a^	2.03 (1.95, 2.32)^a^	1.89 (1.76, 2.23)^a^	2.07 (1.98, 2.37)^a^
	Δ%	1.82 (0.63, 6.61)^a^	13.48 (6.19, 15.55)^b^	0.00 (0.00, 0.47)^a^	0.48 (0.00, 2.18)^a^
Minimum lingual DT	Pre-	1.80 (1.56, 1.95)^a^	1.87 (1.75, 2.13)^ac^	1.51 (1.37, 1.84)^a^	1.73 (1.59, 1.88)^a^
	Post-	1.71 (1.50, 1.86)^a^	1.64 (1.33, 1.76)^a^	1.51 (1.36, 1.84)^a^	1.72 (1.52, 1.84)^a^
	Δ%	4.03 (0.00, 7.49)^a^	14.29 (2.08, 24.78)^b^	0.00 (0.00, 0.00)^a^	2.50 (0.00, 4.09)^a^
Mean proximal DT	Pre-	1.59 (1.40, 1.69)^a^	1.67 (1.54, 1.75)^ac^	1.30 (1.19, 1.47)^a^	1.44 (1,37, 1.52)^ac^
	Post-	1.52 (1.29. 1.61)^a^	1.38 (1.28, 1.48)^a^	1.21 (1.13, 1.43)^a^	1.31 (1.26, 1.43)^a^
	Δ%	6.17 (4.07, 7.56)^a^	16.18 (7.75, 19.88)^b^	4.11 (2.31, 6.40)^a^	5.96 (4.42, 8.24)^a^
Minimum proximal DT	Pre-	1.05 (0.91, 1.18)^a^	1.15 (0.98, 1.28)^ac^	0.86 (0.80, 0.93)^ac^	0.96 (0.81, 0.99)^ac^
	Post-	0.95 (0.76, 1.07)^a^	0.98 (0.70, 1.05)^a^	0.71 (0.67, 0.85)^a^	0.79 (0.68, 0.90)^a^
	Δ%	13.79 (4.63, 20.81)^a^	22.22 (9.88, 30.72)^a^	12.50 (8.43, 18.45)^a^	14.13 (8.06, 22.07)^a^

The 3-D dentin thickness results were shown as median (P25, P75). Different lowercase letters (a or b) in the parameter columns of the same canal third indicate statistical difference between groups. Lowercase letter c indicates a significant difference between the pre-operative and post-operative values. The DT parameters were analyzed using Mann–Whitney test, *P* < 0.05

*DT* dentin thickness, *pre*- the pre-operative parameters, *post*- the post-operative parameters. Δ% percentage change of dentin thickness parameters

Root canal preparation removed certain amount of dentin from all thirds of the root. In the coronal third, the RDT on proximal walls of only 69.2% of teeth was over 1.3 mm in both groups. In the middle third, the RDT of only 38.5% of teeth in oval canal group and 53.8% of teeth in the round canal group remained over 1.3 mm on the proximal walls, while most of the buccal and lingual walls were still thick (over 1.3 mm, 92.3%) (Table 2). The RDT difference between oval canal group and round canal group mainly exist in the proximal dentin wall of the middle third. The proximal dentin wall has significantly lower pre-operative dentin thickness (Fig. 3, Table 2). In addition, more amount of dentin was removed after root canal preparation comparing to buccal and lingual dentin walls in both oval canal group and round canal group (Fig. 3, Additional file 3: Fig. S3, Table2).

In coronal and middle thirds, no significant difference was observed in mean and minimum dentin thickness in all directions (buccal, lingual and proximal) between oval and round canal groups, either before or after root canal preparation (*P* > 0.05) (Table 2). Before root canal preparation, the proximal wall was the thinnest wall, while lingual and buccal wall shared similar thickness in coronal and middle thirds. Regarding the removal of dentin, the lingual wall lost the least amount of dentin thickness in the middle third, whereas the buccal wall lost the least amount of dentin thickness in the coronal third (Table 2, Additional file 3: Fig. S3).

## Discussion

Mandibular incisors have been used to evaluate the efficacy of preparation files in a number of studies [7–12, 14, 18]. To the best of our knowledge, there have been no studies comparing the different preparation outcome of oval and round canals of mandibular incisors, as well as including a separate investigation of each canal third. Therefore, this study highlights the influence of oval canals on preparation process comparing to round canals in mandibular incisors, including detailed investigations on dentin thickness and canal morphological changes in different canal thirds.

Due to the challenging anatomy feature in mandibular incisors, oval canals of mandibular incisors have been studied extensively. Oval canals were selected according to canal AR, but the canal region for AR determination is inconsistent among studies. In the study of Velozo et al., oval canals were determined by the mean AR of all slices 10 mm of apex [14]. Mean AR of slices 6 mm from apex was used to determine oval canals in the study of Azim et al. [12]. In another study of Kaloustian et al., mean AR of coronal two thirds was used to select oval canals from round canals [35]. Micro-CT imaging has been regarded as the gold standard for quantitative and qualitative morphological analyses of root canals. In the present study, the mandibular incisors have similar root canal preparation outcome in the apical third, and the outcome of coronal two thirds were significantly different. This result might suggest that the morphological difference mainly located in the coronal and middle third of the root. Therefore, it is reasonable to determine the AR of mandibular incisors according to the morphological features of coronal two thirds for future studies. However, further studies are needed to support this opinion.

Despite the dissimilarities in root canal morphology (SMI, form factor, AR and roundness), the comparison between the pre- and post-operative canal morphological parameters revealed a significant change in canal shape, indicating a more conical shape was acquired after root canal preparation in both oval and round canal groups. However, significant difference in canal morphological parameters between groups was still observed in the coronal and middle thirds of the canal after root canal preparation. These results may be explained by the non-adaptive feature of rotary files and the canal extension tendency of oval canals toward buccal and lingual direction in the middle and coronal thirds, leaving the extended area ineffectively shaped.

In this study, both oval and round canal groups have left a relatively high mean percentage of UCW (43.55%; 22.70%). More UCW was observed in the oval canal group, which confirms a previous statement that canal morphological variation has more influence on the changes of root canal preparation than the preparation techniques themselves [39]. In each canal third, higher percentage of UCW was only observed in coronal and middle thirds. This further justified the morphological differences between oval and round canals of mandibular incisors mainly exist in the coronal two thirds of the canals, suggesting that the preparation of oval canals of mandibular incisors coronal two thirds should be considered a significant target. Fortunately, Flatsonic and Clearsonic ultrasonic tips were reported to significantly reduce UCW following the creation of a glide path with engine-driven instruments [16, 17]. Therefore, the use of such ultrasonic tips is recommended to aid the mechanical preparation of the canal surface in mandibular incisors.

In the UCW correlation analysis in this study, very strong correlation coefficients were obtained from canal major diameter and canal volume, strong correlation coefficients were also obtained from form factor, canal minor diameter and canal area. Although the influence of canal shape is inconclusive (SMI, roundness moderate correlation, form factor strong correlation), other results demonstrated canal size to be one important factor to cause UCW after root canal preparation. In the review of Siqueira Junior et al., the UCW of different canals in maxillary and mandibular molars were not significantly different [6]. However, the palatal canals of maxillary molars and the distal canals of mandibular molars were reported to be more mildly curved comparing to other canals [45], which suggested that canal volume might be one of the other factors that could influence the UCW in root canal preparation. The influence of canal volume on UCW should be further investigated in future studies.

Extensive dentin removal in the mesio-distal direction after root canal preparation may weaken the fracture resistance of mandibular incisors [21, 28]. In order to balance between infection control and preservation of dentin thickness, sufficient dentin removal without sacrificing tooth resistance during root canal preparation is a challenging task. Silva’s study revealed the greater chance of teeth fracture when the dentin thickness of root was less than 1.3 mm [29]. In this present study, proximal dentin walls were the thinnest and lost most amount of dentin after root canal preparation. This result is in accordance with the previous studies, that proximal dentin wall is originally thinner [46], and more dentin was removed on the proximal dentin wall than buccal and lingual dentin wall after root canal preparation with Ni–Ti files [16].

This study has limitations related to the fact that the teeth were pair-matched based on root canal length while the consistence of the canal size/volume were not considered. Another limitation is the mandibular incisors collected in this study are from the Chinese population. It is possible that the morphological influence on root canal preparation of mandibular incisors might be different in other races. Finally, the sample size of this current study is not big enough to create more experimental groups. Therefore, only one rotary file system, the ProTaper NEXT system, was evaluated in this study due to the sample size limitation. It is possible that adaptive instruments could effectively prepare both oval canals and round canals, but our study could still be beneficial for the non-adaptive file treatment. In order to consolidate the present findings, further studies should be performed considering both root canal length and size, population and different types of Ni–Ti instruments.

Overall, the shaping outcome and the elimination of UCW of the coronal two thirds are more greatly compromised in oval canals comparing to round canals in mandibular incisors, and the preservation of proximal dentin wall should be preferred in both types of canals. In order to achieve a better treatment outcome, the following are suggested to all clinicians: (1) sufficient irrigation should be adopted to enhance chemical preparation, (2) novel ultrasonic tips could be used to reduce the untouched canal area by touching the canal walls in an active mode, (3) the balance of disinfection and proximal dentin preservation should be considered during root canal preparation.

## Conclusions

Within the limitation of this study, the following is concluded:

1. Both types of canals were more conical after root canal preparation, but oval root canals tend to leave more UCW area than round canals in the coronal two thirds of mandibular incisors, which contributes to the challenge for endodontic infection control.
2. In mandibular incisors, the lower dentin thickness and larger amount of dentin removal in the proximal dentin wall were the major concern for the fracture resistance of endodontically treated mandibular incisors.

## Supplementary Information

**Additional file 1:** Figure S1. The schematic presentation of methods for each step of Micro-CT measurement in this study. This study was approved by the school ethics committee. 26 mandibular incisors were pair-matched and grouped. These teeth were prepared and re-scanned by micro-CT. Root canal parameters (SMI, form factor, roundness, AR, major diameter, minor diameter, canal volume, canal area and UCW) and 3D dentin thickness distribution were evaluated.

**Additional file 2**: Figure S2. The root canal morphological parameters in different regions in oval and round canal groups. The values of SMI (A1-3), Formfactor (B1-3), Roundness (C1-3), AR (D1-3), major diameter (E1-3), minor diameter (F1-3), canal area (G1-3) and canal volume (H1-3) before and after root canal preparation in different regions (coronal, middle and apical third). *Indicates *P* < 0.05, **Indicates *P* < 0.01, ***Indicates *P* < 0.001. SMI: Structure model index. AR: aspect ratio.

**Additional file 3**: Figure S3. Percentage change of the 3D dentin thickness parameters after root canal preparation. The change of values of mean dentin thickness (**A**, **E**) and minimum dentin thickness (**C**, **G**) were evaluated in coronal and middle third in both oval and round canal group after root canal preparation. The change percentage of values of mean dentin thickness (**A**, **E**) and minimum dentin thickness (**C**, **G**) were also presented. *Indicates *P* < 0.05, **Indicates *P* < 0.01, ***Indicates *P* < 0.001. SMI: Structure model index. DT: dentin thickness.

**Additional file 4**: Table S1. The root canal morphological parameters in different regions in oval and round canal groups.

## Data Availability

The datasets used and/or analysed during the current study are available from the corresponding authors on reasonable request.

## Abbreviations

Micro-CT: Micro-computed tomography
3D: Three-dimensional
UCW: Untouched canal wall
ROI: Region of interest
AR: Aspect ratio
SMI: Structure model index
RDT: Remaining dentin thickness
