# Supplementary figures and images for: Shaping outcome of ProTaper NEXT for root canal preparation in mandibular incisors: a micro-CT study
Source: BMC Oral Health. 2022 Jul 22;22:302. doi: 10.1186/s12903-022-02335-7 (PMC9308234; doi:10.1186/s12903-022-02335-7)

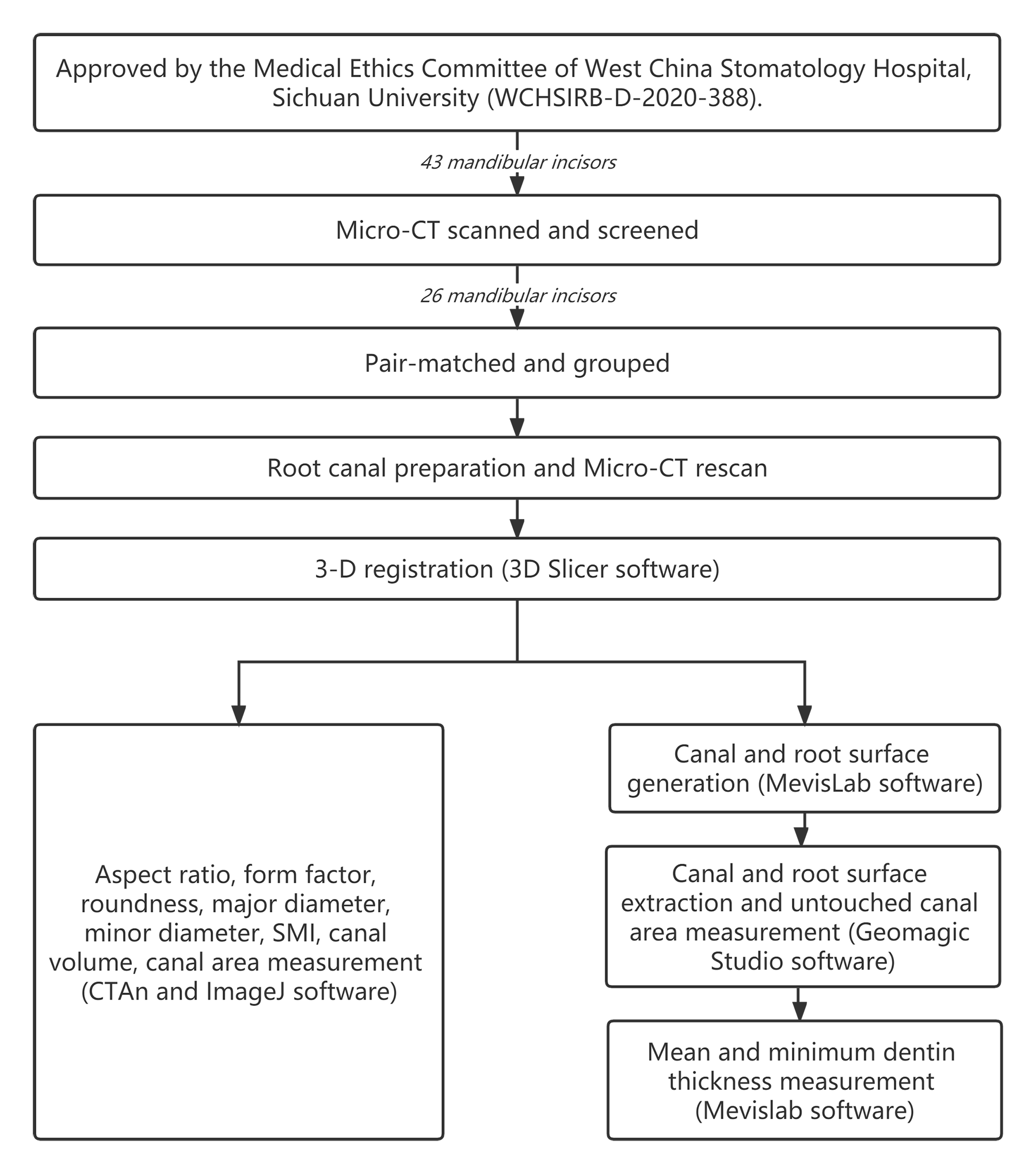

Supplement: Supplementary file 1 — Additional file 1: Figure S1. The schematic presentation of methods for each step of Micro-CT measurement in this study. This study was approved by the school ethics committee. 26 mandibular incisors were pair-matched and grouped. These teeth were prepared and re-scanned by micro-CT. Root canal parameters (SMI, form factor, roundness, AR, major diameter, minor diameter, canal volume, canal area and UCW) and 3D dentin thickness distribution were evaluated. [file 12903_2022_2335_MOESM1_ESM.tif]

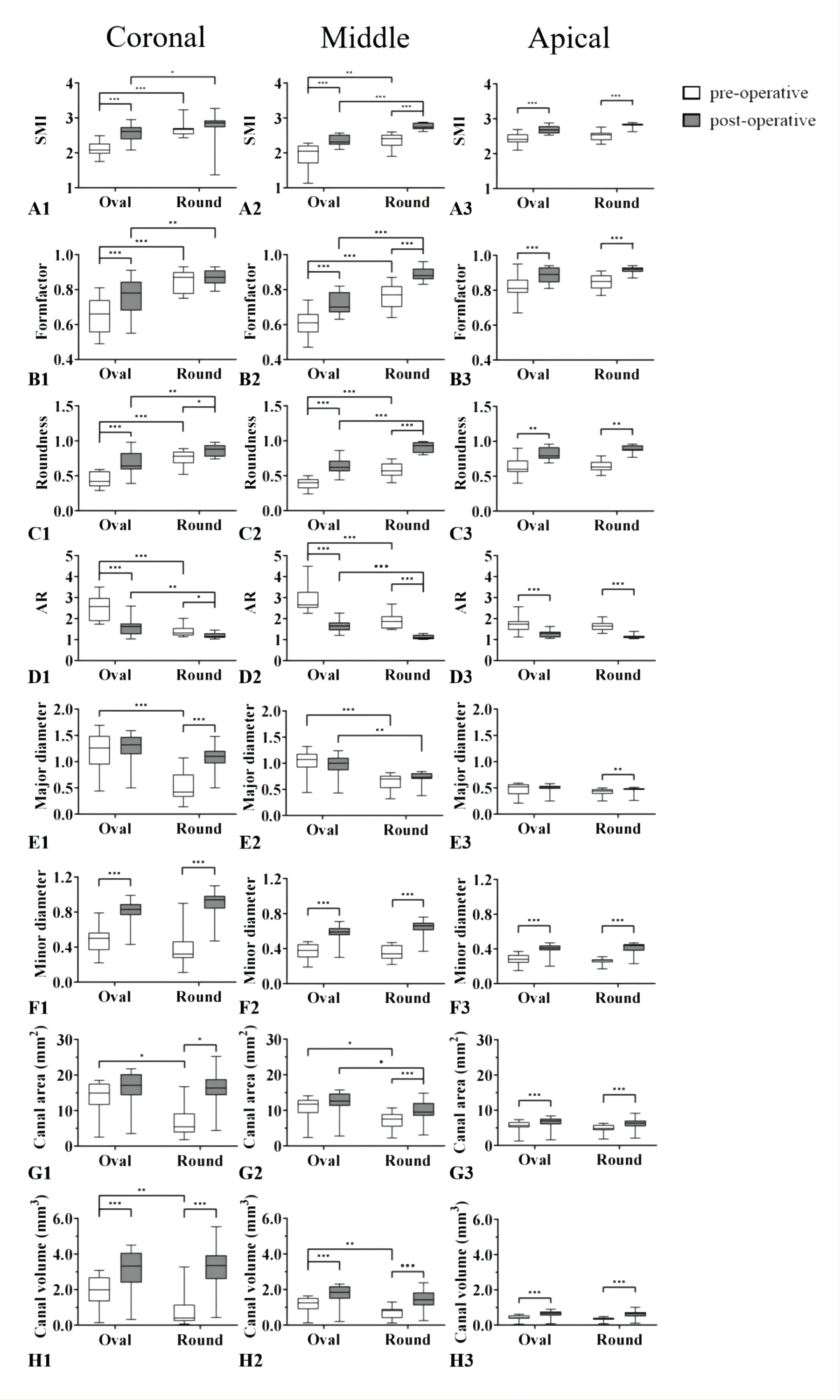

Supplement: Supplementary file 2 — Additional file 2: Figure S2. The root canal morphological parameters in different regions in oval and round canal groups. The values of SMI (A1-3), Formfactor (B1-3), Roundness (C1-3), AR (D1-3), major diameter (E1-3), minor diameter (F1-3), canal area (G1-3) and canal volume (H1-3) before and after root canal preparation in different regions (coronal, middle and apical third). *Indicates P < 0.05, **Indicates P < 0.01, ***Indicates P < 0.001. SMI: Structure model index. AR: aspect ratio. [file 12903_2022_2335_MOESM2_ESM.tiff]

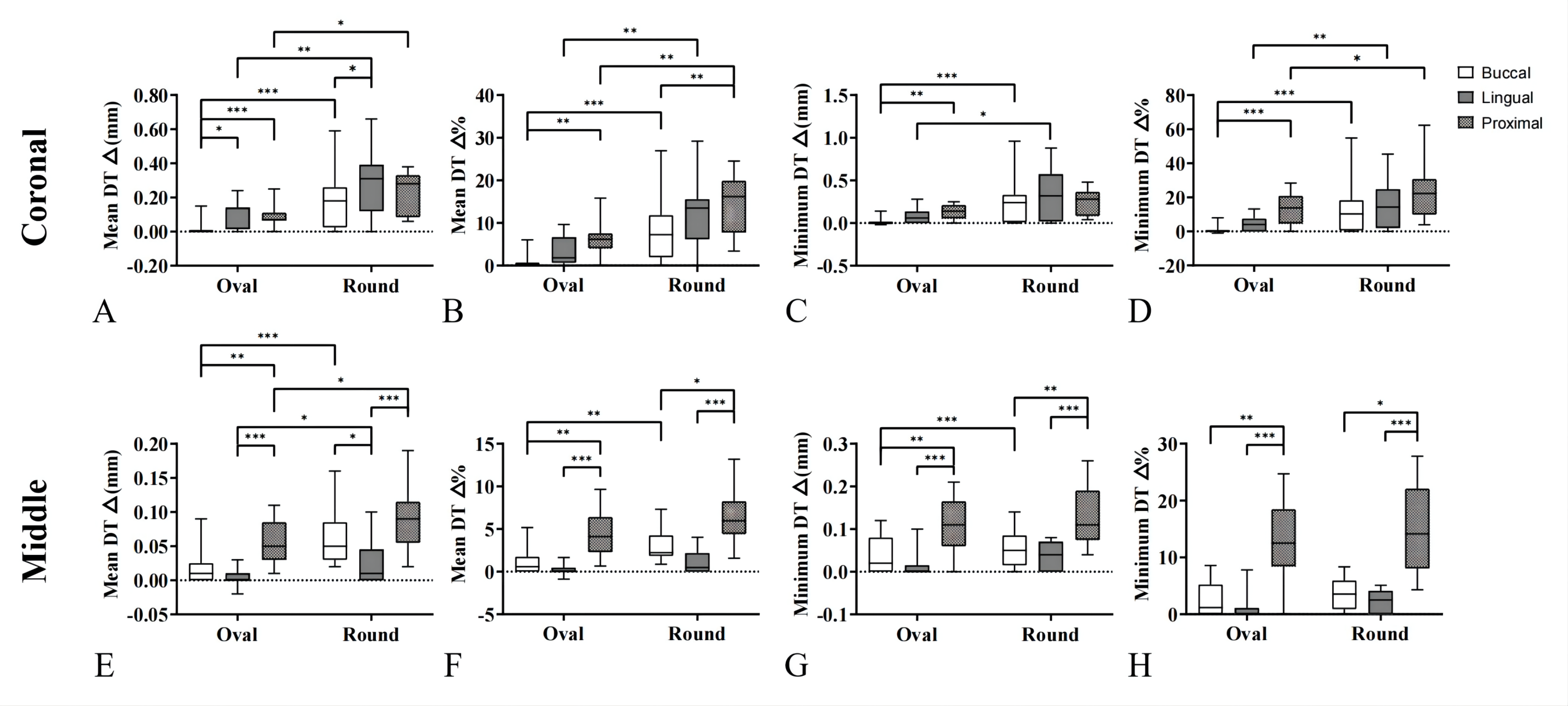

Supplement: Supplementary file 3 — Additional file 3: Figure S3. Percentage change of the 3D dentin thickness parameters after root canal preparation. The change of values of mean dentin thickness (A, E) and minimum dentin thickness (C, G) were evaluated in coronal and middle third in both oval and round canal group after root canal preparation. The change percentage of values of mean dentin thickness (A, E) and minimum dentin thickness (C, G) were also presented. *Indicates P < 0.05, **Indicates P < 0.01, ***Indicates P < 0.001. SMI: Structure model index. DT: dentin thickness. [file 12903_2022_2335_MOESM3_ESM.tiff]
