# Supplementary material for: Shaping outcome of ProTaper NEXT for root canal preparation in mandibular incisors: a micro-CT study
Source: BMC Oral Health. 2022 Jul 22;22:302. doi: 10.1186/s12903-022-02335-7 (PMC9308234; doi:10.1186/s12903-022-02335-7)
Supplement: Supplementary file 4 — Additional file 4: Table S1. The root canal morphological parameters in different regions in oval and round canal groups. [file 12903_2022_2335_MOESM4_ESM.docx]

**Tables**

**Table S1 The root canal morphological parameters in different regions in oval and round canal groups.**

|  |  | **Coronal third** | | **Middle third** | | **Apical third** | | **All thirds** | |
| --- | --- | --- | --- | --- | --- | --- | --- | --- | --- |
|  |  | *Oval* | *Round* | *Oval* | *Round* | *Oval* | *Round* | *Oval* | *Round* |
| UCW | pre- | - | - | - | - | - | - | - | - |
|  | post- | 5.96 (2.29, 7.52)^a^ | 0.17 (0.00, 3.18)^b^ | 4.23 (2.67, 5.90)^a^ | 1.55 (0.31, 2.35)^b^ | 1.78 (0.97, 2.83)^a^ | 1.08 (0.64, 1.81)^a^ | 12.45 (7.77, 16.10)^a^ | 2.81 (1.18, 7.46)^b^ |
|  | △% | 37.82 (23.33, 43.33)^a^ | 4.12 (0.00, 21.68)^b^ | 37.86 (29.16, 44.25)^a^ | 21.68 (5.71, 28.41)^b^ | 34.46 (21.02, 42.48)^a^ | 21.58 (14.15, 34.52)^a^ | 38.59 (32.66, 44.92)^a^ | 17.09 (11.12, 34.11)^b^ |
| SMI | pre- | 2.08 (1.98, 2.27)^ac^ | 2.67 (2.53, 2.71)^b^ | 2.05 (1.70, 2.21)^ac^ | 2.41 (2.21, 2.52)^bc^ | 2.41 (2.33, 2.56)^ac^ | 2.54 (2.39, 2.60)^ac^ | 2.06 (1.89, 2.26)^ac^ | 2.49 (2.33, 2.65)^bc^ |
|  | post- | 2.61 (2.39, 2.74)^a^ | 2.86 (2.73, 2.93)^b^ | 2.31 (2.24, 2.52)^a^ | 2.74 (2.69, 2.87)^b^ | 2.68 (2.58, 2.79)^a^ | 2.83 (2.81, 2.86)^a^ | 2.55 (2.26, 2.68)^a^ | 2.86 (2.73, 2.93)^b^ |
|  | △% | 18.47 (14.79, 27.93)^a^ | 7.43 (2.38, 13.85)^b^ | 17.35 (9.01, 28.90)^a^ | 13.48 (11.75, 25.00)^a^ | 9.39 (6.10, 16.92)^a^ | 13.33 (9.04, 16.67)^a^ | 17.78 (9.14, 29.47)^a^ | 10.24 (0.34, 24.42)^a^ |
| Formfactor | pre- | 0.66 (0.56, 0.74)^ac^ | 0.87 (0.78, 0.90)^b^ | 0.61 (0.56, 0.66)^ac^ | 0.77 (0.70, 0.82)^bc^ | 0.81 (0.79, 0.86)^ac^ | 0.85 (0.81, 0.89)^ac^ | 0.70 (0.64, 0.72)^ac^ | 0.82 (0.80, 0.85)^bc^ |
|  | post- | 0.78 (0.68, 0.85)^a^ | 0.87 (0.84, 0.91)^b^ | 0.70 (0.67, 0.79)^a^ | 0.88 (0.86, 0.92)^b^ | 0.89 (0.85, 0.93)^a^ | 0.92 (0.91, 0.93)^a^ | 0.75 (0.72, 0.85)^a^ | 0.90 (0.87, 0.92)^b^ |
|  | △% | 18.75 (9.06, 24.32)^a^ | -1.09 (-5.13, 4.28)^b^ | 16.18 (10.09, 29.03)^a^ | 12.20 (10.30, 24.71)^a^ | 8.75 (2.42, 14.07)^a^ | 7.06 (5.08, 13.20)^a^ | 12.68 (6.46, 21.58)^a^ | 6.10 (4.14, 11.18)^a^ |
| Roundness | pre- | 0.42 (0.35, 0.57)^ac^ | 0.78 (0.68, 0.85)^bc^ | 0.40 (0.32, 0.45)^ac^ | 0.57 (0.50, 0.68)^bc^ | 0.60 (0.56, 0.73)^ac^ | 0.63 (0.59, 0.71)^ac^ | 0.48 (0.44, 0.53)^ac^ | 0.64 (0.62, 0.72)^bc^ |
|  | post- | 0.64 (0.59, 0.83)^a^ | 0.88 (0.78, 0.94)^b^ | 0.62 (0.57, 0.72)^a^ | 0.93 (0.83, 0.98)^b^ | 0.79 (0.75, 0.92)^a^ | 0.88 (0.87, 0.94)^a^ | 0.69 (0.63, 0.79)^a^ | 0.89 (0.82, 0.94)^b^ |
|  | △% | 61.11 (31.47, 70.71)^a^ | 10.11 (-1.84, 29.61)^b^ | 63.89 (41.67, 99.46)^a^ | 52.31 (43.25, 78.96)^a^ | 32.14 (12.18, 54.12)^a^ | 39.68 (26.86, 52.21)^a^ | 42.86 (29.99, 78.22)^a^ | 32.79 (27.78, 38.10)^a^ |
| Aspect ratio | pre- | 2.57 (1.88, 2.98)^ac^ | 1.31 (1.20, 1.55)^bc^ | 2.65 (2.51, 3.27)^ac^ | 1.86 (1.53, 2.12)^bc^ | 1.73 (1.46, 1.88)^ac^ | 1.64 (1.46, 1.79)^ac^ | 2.31 (2.11, 2.75)^ac^ | 1.68 (1.47, 1.79)^bc^ |
|  | post- | 1.62 (1.26, 1.77)^a^ | 1.15 (1.08, 1.30)^b^ | 1.65 (1.45, 1.82)^a^ | 1.09 (1.02, 1.23)^b^ | 1.29 (1.11, 1.38)^a^ | 1.16 (1.08, 1.17)^a^ | 1.49 (1.31, 1.66)^a^ | 1.17 (1.08, 1.26)^b^ |
|  | △% | 39.39 (24.19, 43.46)^a^ | 8.33 (-1.77, 23.51)^b^ | 38.99 (30.60, 54.08)^a^ | 38.41 (32.56, 45.83)^a^ | 26.20 (14.42, 36.45)^a^ | 29.08 (21.47, 35.69)^a^ | 35.50 (29.95, 47.67)^a^ | 27.52 (24.57, 31.85)^b^ |
| Major diameter | pre- | 1.26 (0.94, 1.49)^a^ | 0.42 (0.33, 0.76)^bc^ | 1.07 (0.92, 1.18)^a^ | 0.70 (0.53, 0.76)^b^ | 0.52 (0.38, 0.57)^a^ | 0.44 (0.39, 0.48)^ac^ | 0.96 (0.76, 1.05)^a^ | 0.54 (0.44, 0.62)^bc^ |
|  | post- | 1.32 (1.14, 1.47)^a^ | 1.10 (0.96, 1.20)^a^ | 1.00 (0.86, 1.11)^a^ | 0.73 (0.70, 0.81)^b^ | 0.52 (0.49, 0.54)^a^ | 0.48 (0.46, 0.50)^a^ | 0.91 (0.84, 1.02)^a^ | 0.77 (0.73, 0.83)^b^ |
|  | △% | -4.10 (-6.25, 15.06)^a^ | 125.32 (17.50, 246.80)^b^ | -5.73 (-8.16, -2.71)^a^ | 11.68 (1.77, 22.54)^b^ | 2.22 (-2.77, 13.72)^a^ | 8.07 (1.21, 14.26)^a^ | -3.22 (-4.76, 7.28)^a^ | 42.22 (12.79, 84.56)^b^ |
| Minor diameter | pre- | 0.50 (0.37. 0.56)^ac^ | 0.32 (0.27, 0.47)^ac^ | 0.38 (0.30, 0.45)^ac^ | 0.34 (0.28, 0.44)^ac^ | 0.28 (0.24, 0.33)^ac^ | 0.27 (0.25, 0.28)^ac^ | 0.38 (0.32, 0.44)^ac^ | 0.31 (0.26, 0.39)^ac^ |
|  | post- | 0.84 (0.77, 0.89)^a^ | 0.94 (0.84, 0.98)^a^ | 0.59 (0.56, 0.64)^a^ | 0.66 (0.61, 0.70)^a^ | 0.41 (0.39, 0.44)^a^ | 0.44 (0.38, 0.46)^a^ | 0.59 (0.57, 0.62)^a^ | 0.67 (0.60, 0.70)^a^ |
|  | △% | 54.14 (41.38, 98.74)^a^ | 161.82 (63.11, 266.35)^b^ | 56.30 (36.31, 95.24)^a^ | 79.58 (52.64, 121.42)^a^ | 37.99 (26.45, 70.68)^a^ | 58.44 (38.75, 80.17)^a^ | 52.72 (41.60, 73.47)^a^ | 129.67 (52.38, 152.82)^a^ |
| Canal area | pre- | 14.93 (11.59, 17.57)^a^ | 5.44 (3.85, 10.59)^bc^ | 11.73 (9.25, 12.92)^a^ | 7.54 (5.42, 8.95)^bc^ | 5.65 (5.11, 6.64)^ac^ | 4.81 (4.34, 5.84)^ac^ | 31.91 (26.12, 32.60)^ac^ | 19.41 (13.60, 21.38)^bc^ |
|  | post- | 17.12 (14.30, 20.18)^a^ | 16.33 (14.35, 18.83)^a^ | 12.59 (11.28, 14.71)^a^ | 9.50 (8.46, 12.05)^b^ | 6.93 (5.96, 7.59)^a^ | 6.35 (5.43, 7.08)^a^ | 34.85 (29.02, 40.86)^a^ | 30.58 (27.64, 35.72)^a^ |
|  | △% | 17.36 (11.64, 32.98)^a^ | 190.23 (32.31, 288.11)^b^ | 11.72 (4.97, 17.98)^a^ | 35.49 (23.22, 58.50)^b^ | 13.27 (7.52, 32.61)^a^ | 24.92 (14.28, 38.10)^a^ | 15.69 (9.93, 27.24)^a^ | 72.75 (31.29, 115.43)^b^ |
| Canal volume | pre- | 1.99 (1.34, 2.69)^ac^ | 0.40 (0.24, 1.16)^bc^ | 1.25 (0.89, 1.53)^ac^ | 0.81 (0.40, 0.91)^bc^ | 0.41 (0.36, 0.55)^ac^ | 0.33 (0.31, 0.42)^ac^ | 3.90 (2.71, 4.10)^ac^ | 1.66 (1.00, 2.35)^bc^ |
|  | post- | 3.32 (2.40, 4.07)^a^ | 3.35 (2.59, 3.93)^a^ | 1.84 (1.48, 2.18)^a^ | 1.42 (1.11, 1.84)^a^ | 0.67 (0.54, 0.77)^a^ | 0.65 (0.50, 0.74)^a^ | 5.80 (4.34, 6.85)^a^ | 5.46 (4.33, 5.90)^a^ |
|  | △% | 46.75 (42.16, 111.81)^a^ | 470.15 (89.02, 1204.28)^b^ | 52.89 (25.67, 65.91)^a^ | 108.33 (67.33, 178.70)^b^ | 50.91 (24.68, 94.95)^a^ | 70.97 (39.58, 124.54)^a^ | 56.18 (37.96, 79.24)a | 241.14 (69.51, 450.95)^b^ |

The root canal morphological parameters before and after canal instrumentation were shown as median (P25, P75). Different lowercase letters (a or b) in the parameter columns of the same canal third indicate statistical difference between oval and round canal group. Lowercase letter c indicates a significant difference between the pre-operative and post-operative values. The canal parameters were analyzed using Mann-Whitney test, *P*< 0.05. UCW: Untouched canal wall. SMI: Structure model index. pre-: the pre-operative parameters. post-: the post-operative parameters. Δ%: the percentage change of the canal morphological parameters.
